# A guide to heat shock factors as multifunctional transcriptional regulators

**DOI:** 10.1111/febs.70139

**Published:** 2025-06-02

**Authors:** Hendrik S. E. Hästbacka, Alejandro J. Da Silva, Lea Sistonen, Eva Henriksson

**Affiliations:** ^1^ Faculty of Science and Engineering, Cell Biology Åbo Akademi University Turku Finland; ^2^ Turku Bioscience Centre University of Turku and Åbo Akademi University Finland

**Keywords:** adhesion, cancer, development, HSE, HSF, HSP, HSR, spermatogenesis, stress, transcription

## Abstract

The heat shock factors (HSFs) form a family of transcription factors, which are evolutionarily conserved in eukaryotes. They are best known as transcriptional regulators of molecular chaperone genes, including those encoding heat shock proteins, in response to heat shock and other protein‐damaging stresses. Since the discovery of the first HSF and its eponymous role in the heat shock response four decades ago, the currently known HSFs in vertebrates, that is, HSF1‐5, HSFX, and HSFY, have been implicated in a wide array of physiological and pathological processes, including organismal development and cancer progression. To date, most studies have focused on individual HSFs, but it is becoming increasingly evident that the role of multiple HSFs and their potential crosstalk should be considered. In this review, we provide a comprehensive overview of the structures, functions, and regulation of the mammalian HSF family members and explore their interplay in biological processes. We highlight recent advancements regarding the roles of HSF family members in viral infection, cell adhesion, and spermatogenesis, and discuss the key questions to be addressed by forthcoming studies in HSF biology.

## Graphical abstract

The heat shock factors (HSFs) are evolutionarily conserved transcription factors best known as regulators of molecular chaperone genes in response to heat shock and other protein‐damaging stresses. Vertebrate HSFs, HSF1‐5, HSFX, and HSFY, are implicated in various physiological and pathological processes, including organismal development and cancer progression. This review provides an overview of the structures, functions, and regulation of mammalian HSF family members and explores their interplay in biological processes.

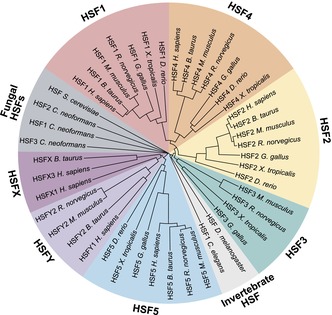

**Abbreviations**
AD: transactivation domain
CAF: cancer‐associated fibroblast
DBD: DNA‐binding domain
EBV: Epstein–Barr virus
ECM: extracellular matrix
HCMV: human cytomegalovirus
HIV‐1: human immunodeficiency virus‐1
HR‐A/B: heptad repeat A/B
HR‐C: heptad repeat C
HSE: heat shock element
HSF: heat shock factor
HSP: heat shock protein
HSR: heat shock response
iPSC: induced pluripotent stem cell
KSHV: Kaposi's sarcoma‐associated herpesvirus
MSA: multiple‐sequence alignment
PDSM: phosphorylation‐dependent SUMOylation motif
PTM: posttranslational modification
RD: regulatory domain

## Introduction

Cells employ protective programs to adapt to stressful conditions that challenge the integrity of their proteome. These programs rely on a large repertoire of molecular chaperones, such as heat shock proteins (HSPs), which prevent the accumulation of misfolded or damaged proteins. Without HSPs aiding misfolded proteins to return to a native state, or directing them to be degraded, cellular functions are hampered, ultimately leading to cell death [1, 2]. The stress source can be external, such as exposure to heavy metals or elevated temperature, but many physiological processes also increase the demand for protein‐folding machinery to maintain protein homeostasis, that is, proteostasis [3]. Indeed, HSPs and other components of the molecular chaperone networks assist in the folding of nascent proteins, their maturation, and proper localization within the cell, even in the absence of stress [4].

**Fig. 1:**
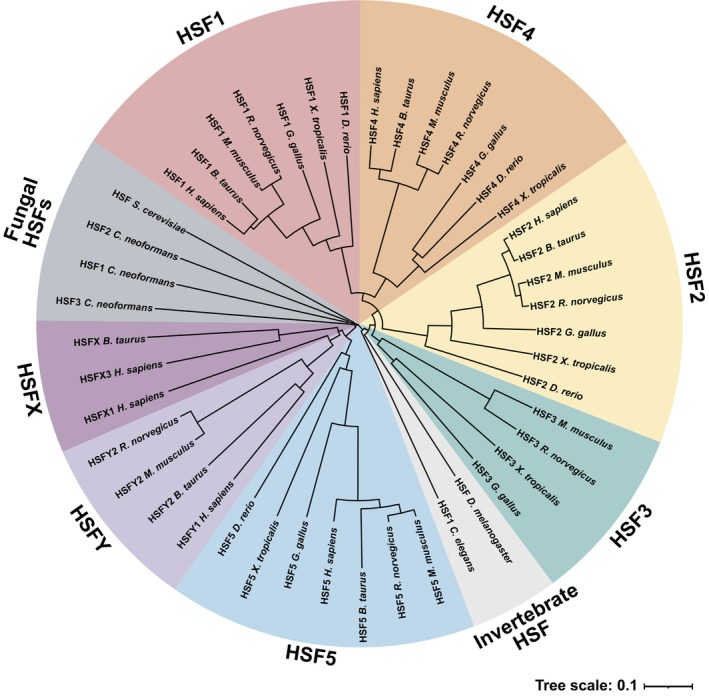
Phylogram based on HSF protein sequences from vertebrates, invertebrates, and fungi, including human (*Homo sapiens*), cattle (*Bos taurus*), mouse (*Mus musculus*), rat (*Rattus norvegicus*), chicken (*Gallus gallus*), western clawed frog (*Xenopus tropicalis*), zebrafish (*Danio rerio*), fruit fly (*Drosophila melanogaster*), fungus (*Cryptococcus neoformans*), and baker's yeast (*Saccharomyces cerevisiae*). Constructed using multiple sequence alignment with ClustalO, phylogenetic tree generation with Simple Phylogeny through EMBL‐EBI Job Dispatcher, and formatted with iTOL [194, 195]. Amino acid sequences were retrieved from the NCBI RefSeq (for *C. neoformans*) or UniProt databases. Tree scale indicates distance; the number of substitutions in proportion to the alignment length, excluding gaps.

**Fig. 2:**
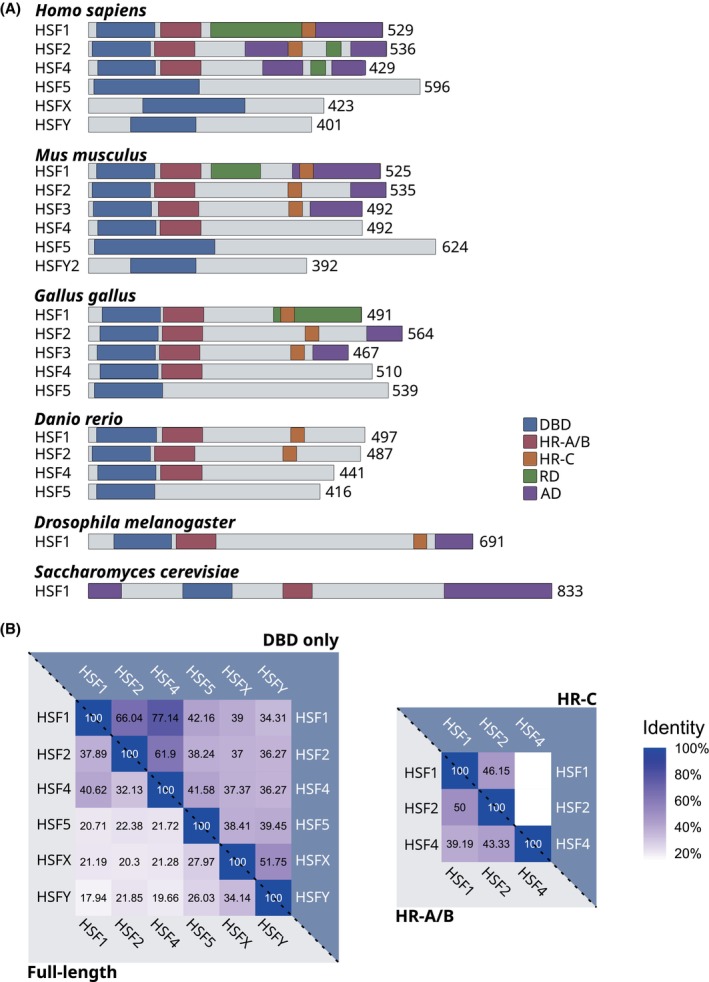
The domain structure of the HSF family is conserved. (A) Comparison of the domain structure of HSFs in human (*Homo sapiens*), mouse (*Mus musculus*), chicken (*Gallus gallus*), zebrafish (*Danio rerio*), fruit fly (*Drosophila melanogaster*) and baker's yeast (*Saccharomyces cerevisiae*). The number of amino acids in each HSF is indicated. AD, transactivation domain; DBD, DNA‐binding domain; HR‐A/B, oligomerization domain; HR‐C, C‐terminal heptad repeat domain; RD, regulatory domain. (B) Sequence identity comparison of human HSFs, determined by multiple‐sequence alignment (MSA) of full‐length HSFs, DBDs, or HR‐domains. EMBL‐EBI Job Dispatcher was used for alignment and percent identity matrices [195]. UniProt accession numbers for the aligned amino acid sequences: Q00613 (HSF1), Q03933 (HSF2), Q9ULV5 (HSF4), Q4G112 (HSF5), Q9UBD0 (HSFX), Q96LI6 (HSFY).

The eukaryotic heat shock factor (HSF) family consists of well‐established transcriptional regulators of HSP genes. HSFs were originally discovered for their role in the rapid induction of HSPs during acute heat stress, a hallmark of the heat shock response (HSR) [1]. The HSR is evolutionarily conserved and is found even in bacteria, such as *Escherichia coli*, where the σ factor σ^32^ performs a similar role as eukaryotic HSFs to uphold proteostasis during stress [5]. Previously, fungi and invertebrates were considered to possess only a single HSF, but multiple HSFs have been identified in certain species, such as in the pathogenic fungus *Cryptococcus neoformans* and the ant *Harpegnathos saltator* [6, 7]. Notably, these recently discovered HSFs and the considerably larger HSF family in plants are functionally related but not orthologous to vertebrate HSFs [6, 7, 8, 9]. The vertebrate HSF family, which according to current knowledge consists of seven members, that is, HSF1‐5, HSFX, and HSFY, expanded early in evolution, which is highlighted by the similarities in their amino acid sequences (Fig. 1). HSF1‐5 are conserved from fish to mammals, but high‐confidence HSFX and HSFY orthologues have only been identified in mammalian species (Ensembl release 113 [10]). The expansion of the family has enabled functional diversification of HSFs (Fig. 2A). The HSFs have proven to be considerably more versatile than their initially described function in the HSR and are associated with a growing list of biological processes [11, 12].

## The HSF family members and their domain structure

Among the HSF family members, HSF1 is known as the master regulator of the HSR and is functionally the counterpart to the single HSF in most yeast and invertebrate species [13]. Consequently, HSF1 is the most studied HSF, and knowledge regarding its function has expanded beyond the HSR to include physiological and pathological processes, such as development, cancer progression, and neurodegenerative diseases [14]. In contrast to the prominent role of HSF1 in the response to acute stress, HSF2 is active in circumstances where the stress is moderate and prolonged [15, 16]. HSF2 is also known to participate in developmental and differentiation‐related processes, including embryogenesis, corticogenesis, and spermatogenesis [17, 18, 19]. While the involvement of HSF1 and HSF2 has been described across different physiological and pathological conditions, the roles of other HSFs are more restricted to specific contexts. HSF4 is best known for being a crucial developmental factor in the eye, and mutations affecting the activity of HSF4 are linked to the occurrence of congenital cataracts in humans [20, 21, 22, 23]. The most recently discovered vertebrate HSF, that is, HSF5, is similarly relevant in differentiation‐related processes, with recent studies focusing on its impact on male fertility [24, 25, 26, 27].

Contrary to the other vertebrate HSFs, functional disparities are evident for HSF3, HSFX, and HSFY. HSF3 is the dominant heat‐responsive HSF in birds, and HSF1 cooperates with HSF3 during the HSR in reptiles [28, 29, 30]. In rodents, HSF3 regulates nonclassical heat shock genes during heat stress, but it is a pseudogene in humans [31]. This demonstrates the variability for HSF3 in vertebrates, and even between mammalian species. Although still understudied, HSFX and HSFY are distinguished from other HSFs by being present as multicopy genes on the X‐ and Y‐chromosomes, respectively [27, 32]. The gene copy number of both HSFs differs among mammals, including variation in the quantity of HSFY genes within a genus [33, 34, 35]. The sex chromosomal HSF gene copies are not all identical in length and may be pseudogenes, as reported for human HSFY [36]. Therefore, in this review, we refer to the human genes corresponding to the longest amino acid sequences, that is, HSFX1 and HSFY1, as HSFX and HSFY.

The members of the HSF family are composed of several domains, which are integral to their activity as transcription factors (Fig. 2A). Some of these domains are structurally highly conserved, but additional domains have been defined based on their functional roles rather than sequence similarity [37]. The highest degree of similarity between HSFs is concentrated in the structured domains, such as the DNA‐binding domain (DBD) or the multimerization domain (HR‐A/B) (Fig. 2B).

The N‐terminal winged helix‐turn‐helix DBD is the defining feature of all the HSF family members. The DBD enables the HSFs to recognize a conserved *cis*‐acting DNA sequence, the so‐called heat shock element (HSE), consisting of inverted repeats of the pentamer 5′‐nGAAn‐3′, where n represents any nucleotide [38]. In contrast to other transcription factors with winged helix‐turn‐helix domains, the wing domain in the HSF DBD does not directly bind to DNA, as evidenced by crystal structures of human HSF1, HSF2, and HSF4 DBDs bound to DNA [23, 39, 40]. Instead, the second helix is inserted into the major groove of the DNA, serving as a recognition helix that binds to the HSE. The differences in structure between the DBDs of HSF1, HSF2, and HSF4 are minor, but sequence analysis of HSF5 indicates that its wing domain is poorly conserved and possibly substituted with a disordered sequence [25, 27]. HSFs display differential affinity to HSEs, to which the organization of the nGAAn pentamers contributes: HSF1 binds longer contiguous HSEs, HSF5 shows preference for short HSEs, and both HSF2 and HSF4 display affinity to discontiguous HSEs [25, 27, 41, 42, 43]. The HSEs are not exclusive to the promoters of protein‐coding genes, but they also direct specific HSFs to drive transcription of noncoding RNAs, such as microRNA and enhancer RNA [44, 45, 46, 47]. The differences between which type of HSE each HSF preferentially binds likely contribute to target their specificity.

HSFs typically contain domains consisting of structurally conserved leucine‐zipper‐like heptad repeats, such as the HR‐A/B and HR‐C domains. The oligomerization domain HR‐A/B, present in HSF1, HSF2, and HSF4, facilitates the assembly of HSF trimers through coiled‐coil interactions between monomers [48]. In addition to their homotrimeric configurations, HSF1 and HSF2 have been found to interact with each other as heterotrimers. The HSF1 and HSF2 HR‐A/B domains have around 50% sequence identity, but key amino acids within the domains are conserved [39, 49] (Fig. 2B). Although complexes composed of HSF2 and HSF4 have also been reported, it is unclear whether the oligomerization domains are involved in these interactions [50]. Notably, HSF5 lacks HR domains but is still capable of oligomerization *in vitro* [27].

**Fig. 3:**
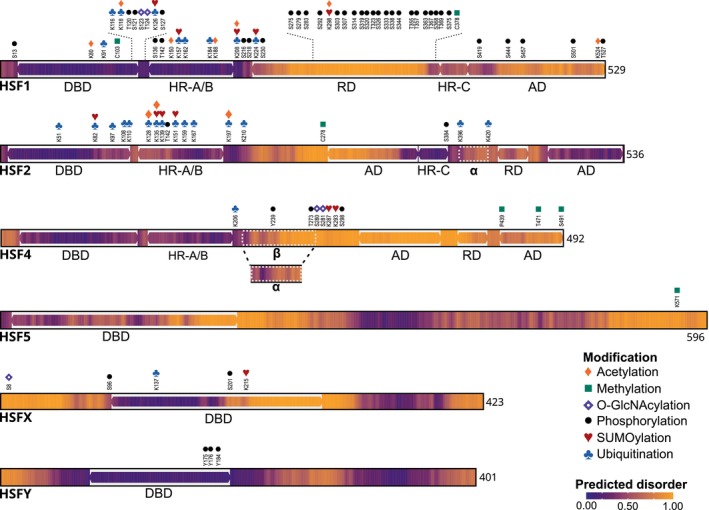
Overview of domains, posttranslational modifications (PTMs) and predicted disorder in human HSFs. The predicted disorder values were determined with AIUpred, with values above 0.5 indicating a likely disordered region [196]. The databases PhosphoSitePlus, iPTMnet, and GlyGen, which include modifications from high‐throughput screens, were used as sources for PTMs [66, 67, 68]. The PTMs of human HSFs are indicated, but additional PTMs have been detected on conserved amino acids of murine HSFs. Acetylation sites on human HSF2 with experimental support have been added [92]. The number of amino acids in each HSF is indicated. AD, transactivation domain; DBD, DNA‐binding domain; HR‐A/B, oligomerization domain; HR‐C, C‐terminal heptad repeat domain; RD, regulatory domain; α, region specific to the α‐isoform; β, region specific to the β‐isoform.

According to our current knowledge, the second leucine‐zipper‐like domain, that is, HR‐C, is specific to HSF1 and HSF2 (Fig. 2A). In the absence of stress, this domain mediates autoinhibition of HSF1 trimerization through interaction with the HR‐A/B domain [51, 52]. Consequently, as HSF4 lacks an HR‐C domain, it assembles into DNA‐binding trimers even in the absence of stress [53]. It is worth pointing out that the currently available databases include HR‐C as a domain in HSF4, likely based on sequence similarity to HSF1 and/or HSF2. However, Nakai and coworkers noted in their original paper on HSF4 that proline residues in key positions distinguish it from an HR‐C domain [53]. This assessment is supported by the model shown in Fig. 3, where the predicted disorder analysis for HSF1 and HSF2 reveals higher structural order in their HR‐C domains, whereas the whole C‐terminal half of HSF4 is predicted to be largely disordered.

The transactivation domains (ADs) in HSF1 serve as interaction sites for cofactors and chromatin remodeling proteins, essential for optimal induction of its target genes during stress [14]. The ADs are repressed by the regulatory domain (RD), keeping HSF1 inactive under normal growth conditions, which enables its activation strictly in a stress‐dependent manner [54]. Domains functionally similar to the AD and RD in HSF1 have been identified in HSF2 and HSF4, but they remain less characterized [55, 56]. Interestingly, two splicing isoforms have been described for both human HSF2 and HSF4 [57, 58] (Fig. 3). The HSF2α isoform contains all 13 exons, but HSF2β lacks a 54‐base pair segment from the AD downstream of the HR‐C, due to the exclusion of exon 11 [59]. While several studies indicate that HSF2α has higher transactivation capacity, the HSF2 isoforms have not been explored comprehensively [11, 57, 60]. In contrast to the exon exclusion in HSF2, the HSF4 isoforms HSF4α and HSF4β arise through alternative splicing of exons 8 and 9 [58]. Both isoforms form trimers and bind to DNA, but since only the HSF4β isoform contains the AD, HSF4α has a repressive effect on the constitutive expression of chaperone genes [58, 61, 62].

## Multilevel regulation of HSF activity

The activity of HSFs is regulated at multiple levels such as gene expression, protein degradation, autoinhibition, interacting partners, phase separation [63], and posttranslational modifications (PTMs). These regulatory layers adjust many aspects of HSF functionality, such as their concentration, oligomerization, DNA‐binding activity, localization, and transactivation capacity [11, 63, 64, 65]. While many of the regulatory mechanisms have been studied primarily in the context of stress, PTMs have been described across diverse conditions, and our knowledge of these modifications is steadily increasing due to high‐throughput screening efforts. The currently known PTMs for HSFs include acetylation, biotinylation, methylation, *O*‐GlcNAcylation, phosphorylation, SUMOylation, and ubiquitination, but many of the modified amino acids have yet to be functionally characterized [62, 66, 67, 68] (Fig. 3). Together, HSFs are regulated by intricate mechanisms to ensure proper activation and attenuation of these transcription factors according to the requirements of the cell.

### Mechanisms of HSF1 activation and attenuation

Since HSF1 was originally discovered as a responsive factor to acute heat shock, its regulatory cycle has been mainly described in this context. Without stress stimulation, monomeric HSF1 molecules remain in an autoinhibitory state, with the HR‐C domain interacting with the HR‐A/B domain, while being associated with a repressive multichaperone complex (e.g., HSPC, HSPA, DNAJ, and TRiC) [65]. When misfolded proteins accumulate during proteotoxic stress, the chaperones in the repressive complex are titrated away from the HSF1 monomers, which in turn are free to trimerize, translocate to the nucleus, and activate gene expression [11]. HSF1 is also capable of sensing stress *in vitro*, since elevated temperatures cause the unfolding of the HR‐C domain, leaving the HR‐A/B free to oligomerize, which occurs in a concentration‐dependent manner [69]. In addition, HSF1 is a target of multiple PTMs such as acetylation, phosphorylation, SUMOylation, and ubiquitination. Upon acute proteotoxic stress, HSF1 is hyperphosphorylated, which correlates with its DNA binding and transcriptional activity [14]. HSF1 phosphorylation‐deficient mutants, however, are still capable of mounting the HSR, and phosphorylation is considered to be a fine‐tuning mechanism for HSF1 activity [70, 71]. Among the many phosphorylation sites in HSF1, S303 stands out as a priming site for the SUMOylation of the adjacent K298, constituting a phosphorylation‐dependent SUMOylation motif (PDSM, ΨKxExxSP, where Ψ denotes a branched hydrophobic amino acid) [72]. Curiously, SUMOylation of K298 inhibits HSF1 transactivation capacity without affecting its DNA binding or trimerization status, suggesting that this modification might disrupt or promote the interaction between HSF1 and transcriptional coactivators or corepressors, respectively [73].

Upon alleviation of proteotoxic stress, HSF1 exhibits a gradual reduction in activity, which is achieved by an HSP‐mediated negative feedback loop and specific PTMs. Once misfolded proteins are restored to their native state or eliminated via degradation, the molecular chaperones become available to once again repress HSF1 [74]. One of the best characterized mechanisms of chaperone‐mediated HSF1 attenuation involves HSPA8 (HSC70), a constitutively expressed member of the HSPA family [75]. Using hydrogen exchange mass spectrometry with purified proteins, Kmiecik and coworkers elegantly showed that HSC70 binds to multiple sites on HSF1 to mediate its attenuation cycle. Notably, the site with the highest affinity corresponds to the AD of HSF1, suggesting that the free pool of HSC70 will initially bind to this domain, triggering a partial attenuation of the HSR. As free HSC70 levels continue to rise, the binding of HSC70 extends to a site in the C‐terminal region of the HR‐A/B and progressively advances toward the N terminus of the trimerization domain, monomerizing HSF1 via an entropic pulling mechanism [75]. Consequently, HSC70‐mediated monomerization of HSF1 follows a sequential process, gradually attenuating the HSR and presumably mediating the recycling of HSF1 monomers.

Although the experiments conducted *in vitro* by Kmiecik *et al*. were performed with an unmodified HSF1 protein, PTMs also play a crucial role in the attenuation process of HSF1 within the cellular context. For example, HSF1 can be dissociated from its target genes upon acetylation of the lysine residues K80 or K118 by the acetyltransferases CBP and EP300, while the acetylation of K208 and K298 enhances HSF1 stability [76, 77, 78]. Moreover, phosphorylation of HSF1 on serine residues S303 and S307 by GSK3β and ERK1, respectively, promotes its interaction with the ubiquitin ligase FBXW7α, leading to its ubiquitination and subsequent proteasomal degradation [79]. It is worth noting that the degradation of HSF1 is of particular clinical relevance, since dysregulation of this mechanism has profound consequences in diseases such as cancer and neurodegenerative disorders. In melanoma, FBXW7α is either mutated or downregulated, correlating with an increase in nuclear HSF1 and cancer progression [79]. Similarly, mutant huntingtin increases the protein levels of FBXW7α, leading to excessive HSF1 degradation in Huntington's disease mouse models, spiny neurons derived from human induced pluripotent stem cells (iPSCs), and patient‐derived brain samples [80]. Taken together, the activation‐attenuation cycle of HSF1 presents several regulatory layers that need to be orchestrated in order to fine‐tune its activity.

### Turnover is an important determinant of HSF2 activity

In contrast to HSF1, the expression of HSF2 varies across different cell types and tissues [81]. The amount of HSF2 correlates with its activity, since HSF2 exhibits high expression and DNA‐binding activity in the mouse preimplantation embryo and human erythroleukemia K562 cells undergoing hemin‐mediated differentiation [82, 83, 84]. Moreover, HSF2 is expressed and capable of binding DNA throughout mouse embryonic development, peaking in the second half of gestation when its expression is restricted to the central nervous system [19]. The cell‐type‐specific expression pattern of HSF2 is also evidenced during developmental processes, such as spermatogenesis and corticogenesis, where HSF2 plays a crucial role [17, 18, 19, 85, 86]. Therefore, HSF2 activity seems to be primarily regulated by abundance, which is congruent with its short half‐life [87, 88]. The levels of HSF2 also vary in a stress type‐dependent manner, underlining the diverse roles of HSF2 in such adverse conditions. Upon heat shock, HSF2 is ubiquitinated and targeted to the proteasome by the APC/C E3‐ligase [87], whereas drastic upregulation of HSF2 occurs during proteasomal inhibition by compounds such as MG132 and Bortezomib [15, 89, 90, 91].

In conjunction with ubiquitination, the stability of HSF2 is governed by acetylation under non‐stress and stress conditions. HSF2 can be acetylated on eight key lysine residues (K82, K128, K135, K197, K209, K210, K395, and K401), of which at least three (K128, K135, and K197) are acetylated by the acetyltransferases CBP and EP300 to inhibit HSF2's proteasomal degradation. Importantly, the influence of acetylation on HSF2 stability has clinical relevance, as patients suffering from a neurodevelopmental disorder characterized by intellectual disability, known as Rubinstein‐Taybi syndrome, exhibit mutations on the CBP or EP300 genes and lower levels of HSF2 due to pronounced degradation [92]. These observations suggest that the control mechanism of HSF2 protein abundance via targeted degradation could also operate during developmental and cell differentiation processes.

Like ubiquitination and acetylation, SUMOylation can regulate the activity of HSF2. For instance, SUMOylation of K82 within the DBD of HSF2 inhibits its DNA‐binding capacity [39]. Intriguingly, a proteome‐wide analysis performed in human cells revealed 20 SUMOylation sites across the HSF2 DBD and HR‐A/B, expanding the range of possible HSF2 modifications, although the precise function of these sites remains unknown [93].

### HSF4, HSF5, HSFX, and HSFY are expressed in a tissue‐specific manner

HSF4 lacks the HR‐C domain and binds constitutively to DNA as a trimer [53]. Similarly to HSF2, HSF4 exhibits a tissue‐specific pattern of expression that includes brain, skeletal muscle, pancreas, and lens of the eye [11]. HSF4 has been extensively studied in lenses due to the striking phenotype of severe cataract in HSF4 knockout mice and the discovery of human HSF4 mutations that lead to the same congenital pathology [20, 22, 94]. Despite the strong functional impact of HSF4 on lens development, its regulatory mechanisms remain poorly understood, with only a few studies shedding light on this topic. For example, phosphorylation of T472 in HSF4β promotes nuclear translocation through interaction with importin β‐1 and enhances the expression of the target genes crystallin alpha B (CRYAB) and HSPB1 (HSP25) [95]. The transcriptional activity of HSF4β has also been shown to be repressed by phosphorylation of S299 within the PDSM and by biotinylation of K444 [62, 96, 97].

HSF5, HSFX, and HSFY have been mostly studied in the context of spermatogenesis, as they were originally found and are preferentially expressed in the male genital tract [98]. At least HSF5 and HSFY are differentially expressed in male germ cells, suggesting that their activity must be carefully regulated during male gametogenesis [27, 32]. For instance, HSF5 is essential for the progression of male germ cells in the meiotic state [27], which is described in more detail in the last section of this review. Forthcoming studies are warranted to elucidate the mechanisms regulating the oligomerization, DNA‐binding, and transactivation capacity of these transcription factors.

## HSFs as multi‐functional transcription factors

**Fig. 4:**
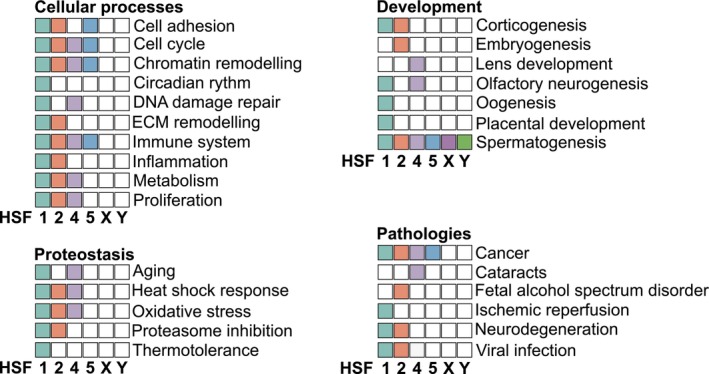
HSFs are involved in a diverse range of physiological and pathological processes. Filled boxes indicate HSFs currently known to be associated with each process. ECM, extracellular matrix. References for HSF functions not mentioned in the main text: Cell cycle and HSF1 [197], HSF2 [190], HSF4 [198], HSF5 [26]; Metabolism and HSF2 [124, 199], HSF4 [138]; Circadian rhythm and HSF1 [200]; Chromatin remodeling and HSF1 [201], HSF2 [202], HSF5 [24]; DNA repair and HSF1 [203]; ECM remodeling and HSF2 [105]; Olfactory neurogenesis and HSF1 [204]; Placental development and HSF1 [136]; Spermatogenesis and HSF4 [183]; Aging and HSF1 [3], HSF4 [205]; Fetal alcohol spectrum disorder and HSF2 [188]; Ischemic reperfusion and HSF1 [206, 207].

Since the discovery of HSFs as regulators of HSPs during acute heat stress, knowledge of their biological functions has markedly expanded. This includes protection against other types of proteotoxic insults and association with multiple physiological and pathological conditions [11] (Fig. 4). The protection against acute proteotoxic stresses is primarily conferred by HSF1, but cells lacking HSF2 display poorer survival when exposed to prolonged moderate heat stress [16, 99]. Cells deficient in either HSF1 or HSF2 are also more sensitive to stress caused by pharmacological inhibition of HSP90 or the proteasome [15, 89, 100]. HSF1 and HSF2 have been demonstrated to share binding sites on stress‐related genes, including HSPs, during heat‐induced and oxidative stress [44, 101, 102]. Although HSF2 can modulate the stress‐inducible expression of target genes, this effect is HSF1‐dependent [57, 102, 103, 104].

HSF2 is activated by temperatures in the febrile range (38–41 °C), rather than by severe heat stress. At higher temperatures, HSF1 activity increases, while the activity and protein levels of HSF2 decrease [16, 87, 101]. HSF2 knockout mouse embryonic fibroblasts display HSF1 activity at lower temperatures, indicating that HSF2 may have a role in modulating HSF1 activation dynamics [105]. Interestingly, the temperature required for HSF1 activation is lower in male germ cells, which has been demonstrated in mice (35–38 °C) and rainbow trout (22 °C) compared to the activation temperature in the respective somatic cells (42 °C and 28 °C) [106, 107]. The specific expression of HSF2, HSF5, HSFX, and HSFY in testes and male germ cells suggests that the activation threshold of HSF1 may be influenced by these other members of the HSF family.

The activation of HSF4 is stress‐independent, and this factor is mainly known as a regulator of constitutively expressed chaperones, such as CRYAB and HSPB1 (HSP25), which are critical for lens development [14, 58, 94]. HSF4 also drives the expression of the antioxidase heme oxygenase 1 (HMOX1) in human lens epithelial cells by binding the proximal HSE on the gene promoter, indicating that HSF4 may influence the cellular redox balance [108]. The same HSE is bound by HSF1 during heat shock, which induces HMOX1 expression [109]. HSF4 is not required for the HSR but participates by inducing nonclassical heat shock genes and by directing chromatin modifications that modulate HSF1 occupancy under stress conditions [110]. In contrast, HSF5 was reported to be dispensable for the HSR in mouse testes, although it can form multimers in a heat‐responsive manner *in vitro* [27]. Additionally, neither the expression nor subcellular localization of HSFY changes in response to heat shock in mouse testes or epididymis [111]. These results suggest that HSF5 and HSFY may not be directly linked to acute heat shock, but a role in responses to other stresses, such as oxidative stress, cannot be excluded.

### Dysregulation of HSFs is associated with malignancies

Given the important roles of HSFs in development and stress responses, it is not surprising that they are also implicated in multiple pathologies (Fig. 4). Disturbances in proteostasis are intimately involved in cancer progression, where factors such as genetic instability, high protein synthesis, and an excess of reactive oxygen species impair the balance between protein production, folding, and degradation. The levels of HSF1 and HSPs are frequently elevated across different cancer types, correlating with poor prognosis [112, 113]. HSF1 is linked to tumorigenesis, as demonstrated by marked suppression of tumor occurrence in mouse carcinogenesis models where HSF1 expression was disrupted [114, 115]. However, the cancer‐associated transcriptional program driven by HSF1 is not limited to HSPs, but includes genes involved in cell proliferation and adhesion, as well as development and metabolism [113]. HSF1 further supports malignancy by non‐cell‐autonomous mechanisms, where it regulates gene expression in cancer‐associated fibroblasts (CAFs) to alter the tumor microenvironment by extracellular matrix (ECM) remodeling and by the secretion of signaling molecules that promote cell proliferation [116, 117, 118]. Importantly, the transcriptional programs of HSF1 in acute stress, cancer cells, and CAFs are distinct from one another [113, 118].

In addition to the extensive involvement of HSF1 in cancer, both HSF2 and HSF4 have been shown to affect cancer cell proliferation and invasion, and altered expression of HSF5 is associated with several cancer types [15, 102, 119, 120, 121]. In contrast to HSF1, which promotes tumor progression, the effects of other HSFs on cancer are more diverse. HSF2 was abnormally expressed in 25 out of 33 human cancers in datasets from the cancer genome atlas program, but was linked to either favorable or poor overall survival depending on the cancer type [122]. This context‐dependent role of HSF2 in cancer is supported by several studies, as HSF2 has been reported to promote proliferation in different cancers, including hepatocellular carcinoma and breast cancer, but can also act as a suppressor of prostate cancer invasion [102, 120, 123, 124]. The roles of HSF1 and HSF2 in cancer have been explored more in depth in other reviews [125, 126].

Similar to HSF1, knocking out HSF4 delayed tumor development and cancer cell proliferation in a carcinogenesis mouse model [127]. The HSF4 expression levels in human hepatocellular carcinoma and colorectal cancer datasets correlate with poor prognosis, but the underlying mechanism is yet to be elucidated [121, 127, 128]. However, HSF4 contributes to the repair of DNA strand breaks by promoting the expression of the recombinase RAD51, a direct interactor of BRCA2 DNA repair‐associated protein [129, 130]. HSF4‐mediated RAD51 expression has not been evaluated in cancer, where elevated RAD51 expression is linked to poor prognosis [131, 132], but has been demonstrated to counteract the accumulation of cataract‐promoting DNA damage in the lens [129]. Interestingly, while HSF5 is most prominently expressed in testes, it is reportedly downregulated in several cancer types and correlates with poor patient survival in lung adenocarcinoma [119]. The altered expression of HSF5 in cancer was also associated with gene expression changes in the immune response and inflammatory signaling pathways. HSF1, HSF2, and HSF4 have been similarly linked to these pathways, suggesting that the HSFs may have common target genes or belong to common gene regulatory networks [133, 134, 135, 136, 137, 138].

### Viruses co‐opt HSF‐mediated transcriptional regulation

In addition to their roles in other pathology‐associated processes, HSFs are involved in viral infections. HSF1 has been identified as a key host factor for the successful completion of the infection cycle of orthopoxviruses, for example, the viruses that cause monkeypox and smallpox [139]. Viral infection leads to HSF1 activation and consequently an increase in the expression of HSP genes, such as members of the HSPA, DNAJ, and HSPC families, which support the assembly and release of viral particles. A recent study showed that HSF1 has a similar role in human coronavirus infection, including SARS‐CoV‐2 variants and low‐pathogenic seasonal coronaviruses [140]. The replication of both orthopox‐ and coronaviruses can be perturbed by pharmacological inhibition or RNAi‐mediated silencing of HSF1.

In human immunodeficiency virus‐1 (HIV‐1) infection, HSF1 is involved in both HIV‐1 transcription and reactivation [141]. Notably, HIV‐1 infection of CD4^+^ T cells is associated with an increase in the abundance of HSF1 due to corecruitment of HSF1 and the viral protein negative factor (Nef) to an HSE located on the HSF1 promoter. The Nef‐HSF1 interplay induces transcription of the HSF1 gene, which likely contributes to the elevated HSP levels observed in HIV‐1 infected cells [141, 142]. However, the function of HSF1 in HIV‐1 reactivation is more contentious, as several studies have reported that HSF1 either mediates or represses HIV‐1 reactivation [141, 143, 144, 145]. Nekongo *et al*. have suggested that the disparity between results may be due to differences in methodology for modulating HSF1 expression or activity in each study. The impact of HSF1 on viral infections, including HIV‐1, was comprehensively described in a recent review [146].

HSF1, HSF2, and HSF4 have been linked to several herpesviruses, especially human cytomegalovirus (HCMV) and the oncogenic gamma‐herpesviruses Epstein–Barr virus (EBV) and Kaposi's sarcoma‐associated herpesvirus (KSHV) [97, 147, 148, 149]. The life cycles of herpesviruses are biphasic, consisting of a latent phase where the virus is dormant, as well as a lytic phase during which viral replication, assembly, and release occur [150]. Notably, several genes expressed during the lytic phase are associated with carcinogenesis [150]. HSF1 is activated in cells infected with HCMV, promoting host cell survival [149], and a recent study reports that pharmacological inhibition of HSF1 reduces the expression of HCMV genes [147]. These results indicate that HSF1 may be important for the infection and replication cycles of HCMV. While the impact of HSF4 on HCMV infection is not known, experiments with recombinant adenoviral particles have demonstrated that HSF4 is able to directly bind an HSE on the CMV immediate‐early promoter, a strong promoter commonly used in plasmids for ectopic gene expression [97]. The HSF4 occupancy has a repressive effect on the CMV promoter, which is specific for the HSF4β isoform, as its SUMOylation depends on phosphorylation of S299 in the PDSM, which is not present in the HSF4α isoform.

HSF1 and HSF2 have been suggested to regulate EBV and KSHV gene expression [148, 151], and the replication of both viruses is dependent on chaperones from the HSP70 family [152, 153]. In EBV‐infected marmoset B cells (the B95‐8 cell line), transcription of the latency‐establishing EBV nuclear antigen 1 gene is induced by heat‐activated HSF1 through direct binding to an HSE within the EBV latency promoter [151]. It was recently reported that HSF2 drives the expression of lytic genes in both EBV and KSHV [148]. This study indicates that HSF2 binds directly to an HSE on the promoter of the lytic cycle transcription factor RTA, encoded by ORF50. Interestingly, HSF2 occupies the promoter specifically during latency, resulting in subtle expression of downstream lytic genes during the latent phase independently of HSF1. As several lytic genes are associated with tumorigenesis [150], Cutrone *et al*. [148] suggest that HSF2 could be a pro‐oncogenic factor in KSHV‐ and EBV‐infected cells by promoting expression of lytic oncogenes in latently infected cells.

To date, no involvement of HSF5, HSFX, or HSFY in viral infections has been reported. This may be due to their predominant expression in the male germ cells, where they are protected by the blood–testis barrier. However, certain viruses can cross the blood–testis barrier, such as hepatitis B virus and Zika virus, which can then infect both Sertoli and germ cells. Consequently, a putative role of HSF5, HSFX, and HSFY in viral infections cannot be disregarded [154].

### Emerging roles of HSFs in cell adhesion and ECM remodeling

The process of cell adhesion is fundamental for multicellular organisms to orchestrate development, maintain tissue integrity, and respond to mechanical stimuli [155, 156, 157]. During tissue morphogenesis, cells must be physically connected to each other (cell–cell adhesion) and to their substrate (cell–matrix adhesion) in order to regulate their size, number, and shape [158]. A striking example is corticogenesis, where postmitotic neurons rely on cell adhesion to migrate, proliferate, and connect with each other to form intricated circuits throughout different neuronal layers [159]. Mechanical forces transmitted through cell adhesion structures constitute crucial signals during embryogenesis to regulate processes such as cell death and differentiation [155]. As a consequence, defective cell adhesion leads to drastic developmental defects and pathologies such as immunological disorders, cardiovascular diseases, and cancer [160, 161]. Remarkably, HSF1 and HSF2 have been linked to cell adhesion through a variety of unbiased proteomic and transcriptomic screens, leading the field into new and exciting research directions.

Although HSF1 is best known for its role as the master regulator of the HSR, this versatile transcription factor also induces the expression of cell adhesion genes in pathological and stress conditions [44, 102, 118]. In highly malignant cancer cells, HSF1 has been shown to drive a transcriptional program distinct from heat shock, which includes cell‐substrate adhesion receptors called integrins and components of the ECM [113]. Congruently, reduced levels of HSF1 in breast cancer cells impair cell adhesion to collagens, reducing cell motility [162]. The effect of HSF1 on adhesion genes is not restricted to only cancer cells, as HSF1 operates in a similar manner in CAFs [116, 117, 118]. Using an elegant experimental design, Scherz‐Shouval *et al*. unveiled that CAFs lacking HSF1 failed to induce the expression of genes related to ECM organization and adhesion in cocultured cancer cells, when compared to their WT counterparts [118]. Since this seminal study, the role of HSF1 in the tumor microenvironment has been expanded to CAFs of breast, colon, and gastric cancers, where it regulates genes encoding ECM components to enhance cancer progression [116, 117, 163]. In response to stress, HSF1 regulates similar types of adhesion‐related genes. For example, upon treatment with geldanamycin, an HSP90 inhibitor that induces the HSR, HSF1 binds to the promoter of fibronectin and induces its transactivation [164]. Upon heat shock, HSF1 was recently shown to indirectly modify the expression of genes involved in cell‐matrix adhesion by binding to enhancers, which are distal regulatory elements that promote gene expression through chromatin conformational changes [44, 165]. It is therefore important to consider that multiple mechanisms orchestrate the HSF1‐dependent control of cell adhesion [44, 47, 165].

In contrast to HSF1, HSF2 appears to influence cell adhesion primarily by regulating the expression of transmembrane adhesion receptors rather than ECM components. For instance, HSF2 has been shown to affect the expression of different adhesion‐related genes, including members of the cadherin superfamily [15]. The cadherin superfamily includes a variety of calcium‐dependent transmembrane receptors that mediate cell–cell adhesion, among which E‐ and N‐cadherins are the best characterized for their roles in development and cancer [159, 166]. At least one member of each cadherin subfamily is downregulated in the absence of HSF2 under physiological conditions, providing a plausible explanation for the mispositioning of neurons in the cortical layers of HSF2 knockout mice [15, 18]. Interestingly, a study conducted with cells derived from patients suffering from Rubinstein–Taybi syndrome demonstrated that these cells exhibit reduced levels of HSF2 and impaired N‐cadherin expression [92].

In addition to regulating cell adhesion genes, HSF2 interacts with several adaptor proteins that link adhesion receptors to the cytoskeleton, such as catenin delta 1 (CTNND1), zonula occludens 1 (ZO1), zonula occludens 2 (ZO2), and talin 1 (TLN1) [167]. Recently, a detailed immunohistochemical analysis of HSF1 and HSF2 expression in a large cohort of benign human tissue samples revealed that HSF2 is present at cell–cell adhesion sites in the liver, epididymis, and cardiac muscle [168]. Among these tissues, the cardiac muscle is particularly interesting since HSF2 was found at specialized adhesion structures called the intercalated disks. The intercalated disks facilitate rhythmic heart contractions by providing strong cell–cell contacts between cardiomyocytes and contain a variety of adhesion proteins, including CTNND1, ZO1, ZO2, and TLN1 [169, 170]. Based on this observation, it has been hypothesized that HSF2 acts as a damage sensor for cell–cell contacts and is transported from the membrane to the nucleus to activate gene expression upon mechanical strain [168, 171]. To test this hypothesis, it is crucial to identify the proteins that keep HSF2 sequestered at cell–cell adhesion sites, disrupt those interactions, and evaluate the outcome upon mechanical stimuli. Remarkably, CTNND1, ZO1, ZO2, and TLN1 are known to localize in the nucleus and influence gene expression, indicating that HSF2 could participate in the constant dialog between the plasma membrane and the nucleus through these protein–protein interactions [167, 172, 173].

Taken together, HSF1 and HSF2 play a role in cell adhesion under both physiological and stress conditions by regulating the expression of key adhesion‐related genes, including ECM components and transmembrane adhesion receptors. This function might not be restricted to these two HSFs, since elevated levels of HSF5 correlate with the upregulation of adhesion genes upon differentiation of neuronal stem cells into central nervous system cell lineages [174]. Future studies are therefore required to unveil whether other HSF family members are also involved in cell adhesion.

### Spermatogenesis highlights interplay of HSFs in developmental and cell differentiation programmes

**Fig. 5:**
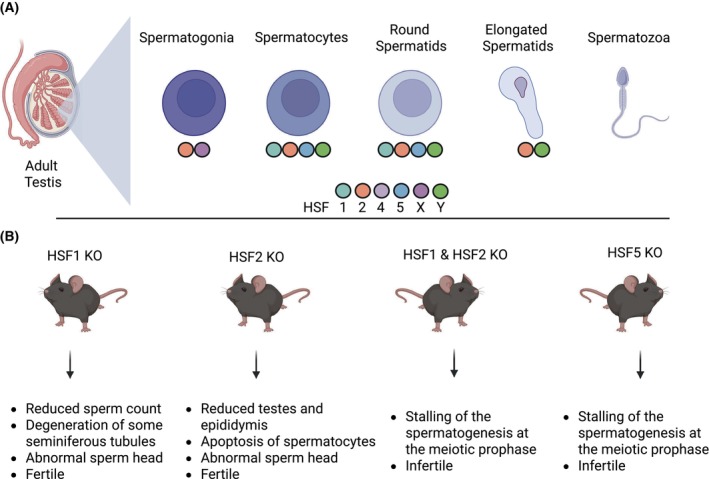
HSFs in spermatogenesis. (A) Expression patterns of mouse HSF1, HSF2, HSF4, HSF5, and HSFY in spermatogonia, spermatocytes, round spermatids, elongated spermatids, and spermatozoa [27, 111, 177, 178]. The expression pattern of HSFX corresponds to human, from Human Protein Atlas proteinatlas.org [81], since mouse data is not currently available. (B) Summary of phenotypes corresponding to knockout HSF1, HSF2, HSF1 & HSF2, and HSF5 knockout mice [27, 85, 136, 180]. Created in BioRender [208].

HSFs play critical roles in different developmental processes, such as embryogenesis, corticogenesis, and spermatogenesis [18, 19, 85]. In particular, spermatogenesis is a striking example, since most members of the HSF family are required for sperm production. Through numerous cell division events involving mitosis and meiosis, the male germ cells mature in specialized structures called the seminiferous tubules. Continuous waves of cell differentiation give rise to the following cell types in order of differentiation: spermatogonia, spermatocytes, postmeiotic round spermatids, and spermatozoa [175, 176] (Fig. 5). In mice, HSF1 is predominantly expressed in spermatocytes and round spermatids, while HSF2 has been detected in all spermatogenic cells except mature spermatozoa [17, 177, 178]. Similarly, HSF5 is predominantly expressed in pachytene spermatocytes and round spermatids, whereas HSFY is present in spermatocytes as well as round and elongating spermatids [27, 32].

Mice lacking either HSF1 or HSF2 exhibit similar disruptions in spermatogenesis, such as increased apoptosis of spermatocytes and morphological defects in sperm heads, but they remain fertile [17, 85, 177, 179]. However, HSF1 and HSF2 double knockout mice are infertile, due to stalling of spermatogenesis in the spermatocyte state [180]. This drastic phenotype in the double knockout mice suggests synergistic transcriptional activity between HSF1 and HSF2, as both factors co‐occupy specific regions of the chromatin in mouse spermatocytes [101]. Furthermore, HSF1 and HSF2 have been shown to interact in mouse testes under physiological conditions and directly regulate Y‐chromosomal multicopy genes that are essential for meiosis and correct packing of the chromatin in sperm heads [17, 49, 177, 181]. There is therefore compelling evidence for a functional interplay between HSF1 and HSF2 during spermatogenesis, likely through their heterotrimerization [98, 101].

Although the HSF5 protein was initially detected in rat and mouse testes, the involvement of this transcription factor in spermatogenesis was demonstrated using a zebrafish knockout model [26, 182]. HSF5 knockout fish display male infertility due to drastically reduced sperm count and defects in sperm morphology, which were attributed to transcriptional dysregulation of cell cycle and apoptosis genes [26]. Only recently have HSF5 knockout mice been generated [24, 25, 27]. Similar to HSF1 and HSF2 double knockouts, mice lacking HSF5 exhibit stalling of spermatogenesis at the spermatocyte stage, leading to male infertility [27]. However, HSF5 binds to a set of genes that are fundamentally different from those occupied by HSF1 and HSF2 in male germ cells under physiological conditions. This difference in target gene specificity can be explained by the HSF5 affinity for shorter HSEs as compared to other HSFs [27]. Curiously, HSF5 prominently occupies the promoter of HSF2 in mouse spermatocytes, suggesting that it could regulate the expression of other HSFs in this particular cell type [25]. Regarding the other HSFs, our knowledge of HSF4, HSFX, and HSFY in spermatogenesis is scarce. Nevertheless, disrupted expression of HSF4 and HSFY has been associated with infertility, while the impact of HSFX on male germ cell differentiation is not known [32, 36, 183].

It is important to note that the process of spermatogenesis is extremely vulnerable to temperature elevation, which causes extensive DNA damage and massive apoptosis of spermatocytes [184]. While HSF1 is crucial for the survival of the more immature germ cells, it appears to have a divergent role in spermatocytes. Intriguingly, spermatocytes in HSF1 knockout mice are protected against cell death upon heat exposure or doxorubicin‐induced genotoxic stress, indicating that HSF1 functions as a quality control factor in these male germ cells through a mechanism that is different from the HSR [179, 185]. In agreement, mice expressing an active form of HSF1 are sterile due to arrest of spermatogenesis at the meiotic stage, and the phenotype of these mice is similar to that observed in WT mice exposed to a single heat shock [186]. Since HSF1 and HSF2 cooperate during spermatogenesis, it is also important to consider a plausible interplay of both transcription factors under stress conditions in male germ cells. Korfanty *et al*. studied the chromatin occupancy of HSF1 and HSF2 in mouse spermatocytes upon mild (38 °C) and severe (43 °C) hyperthermia. While HSF2 genome‐wide binding decreases progressively upon temperature elevation, HSF1 exhibits a distinct pattern of chromatin occupancy by decreasing at 38 °C and peaking following exposure to 43 °C [101]. In contrast to physiological conditions, HSF1 and HSF2 do not seem to synergize under heat stress in male germ cells, evidencing their individual functions. To date, HSF5 and HSFY have not been thoroughly studied in the testes during hyperthermia. However, HSF5 appears to be dispensable for the HSR, as HSF5 knockout testes show a similar pattern of differentially expressed genes compared to their heterozygous counterparts when exposed to 33 °C versus 37 °C *ex vivo* [27]. Taken together, most HSF family members coexist in spermatogenic cells, displaying a distinct, cell‐type‐specific expression pattern throughout the differentiation program.

## Future perspectives and concluding remarks

Forty years ago, the first HSF was identified in fruit fly as a transcription factor that, upon acute heat shock, bound a consensus sequence in the promoters of HSP genes to induce their expression [187]. Since then, the HSFs have been established as versatile transcription factors involved in a wide range of cellular processes, such as development, responses to various types of stress, viral infection, and pathologies. Extensive transcriptomic data and chromatin occupancy profiles have revealed that HSFs regulate distinct gene expression programs depending on the biological context [15, 17, 25, 27, 44, 101, 102, 113, 118, 177, 188, 189, 190, 191]. How these factors identify their target genes under different conditions remains as an outstanding question. In this regard, the discovery that HSF1 and HSF2 regulate distinct sets of genes through enhancers, as compared to promoters, represents a major advancement in the field [44, 47]. Currently, this phenomenon has been predominantly described under stress, and future studies are needed to explore whether it extends to developmental or pathological circumstances. Other critical regulatory factors influencing the activity of HSFs include their interacting partners and PTMs. Some interactome studies have characterized specific protein networks for HSF1, HSF2, and HSF5 in different conditions, but the interacting partners of other HSF family members remain largely unexplored [167, 192, 193]. Likewise, the currently known spectrum of PTMs for HSF1 is significantly broader than for other HSFs (Fig. 3), and our insights come primarily from malignant cancer cells with intrinsic anomalies in signaling cascades. Therefore, studies comparing the protein networks and PTM patterns of all HSFs across distinct biological milieus, such as development, proteotoxic stress, neurodegenerative diseases, and cancer, will be crucial to understand the functional dynamics and synergistic interactions of these transcription factors.

HSF biology has thus proven to be more complex than initially anticipated, as reflected by the diversification of HSFs in vertebrates (Figs 1 and 4). Their physiological and clinical relevance is particularly noticeable in processes such as sperm production and cancer progression, in which most human HSFs are involved. Considering the significant consequences of dysregulation of HSFs, a fundamental question arises: Are the HSFs part of common regulatory networks in these biological processes? The emerging role of HSF1 and HSF2 in cell adhesion provides a compelling framework to explore this question. Indeed, HSF1 plays a key role in regulating ECM architecture, while HSF2 governs the expression of transmembrane adhesion receptors and interacts with adaptor proteins that link adhesion structures to the cytoskeleton [15, 116, 117, 118, 167, 168]. Similarly, HSF5 has been associated with the expression of adhesion proteins upon neuronal differentiation. These findings strongly suggest that the developmental defects and cancer‐related phenotypes linked to HSFs might arise from impaired or dysregulated cell adhesion and tissue architecture.

In conclusion, although many facets of HSF biology remain to be elucidated, substantial milestones for resolving their function and regulation have been reached. As our understanding increases, it is strikingly evident that different members of the HSF family exhibit both specialized and complementary roles, which can vary depending on the tissue and cell type. In this regard, advancements in single‐cell and spatial omics offer powerful means to unveil the molecular mechanisms of the HSFs in their physiological context. Without a doubt, the field of HSF research is gaining strong momentum, propelling it toward an exciting new era.

## Conflict of interest

The authors declare no conflict of interest.

## Author contributions

HSEH, AJDS, LS, and EH conceptualized the review. HSEH performed analysis and visualization of protein disorder. HSEH and AJDS wrote the original draft. HSEH, AJDS, LS, and EH wrote the revised manuscript.
